# Endothelial Senescence Drives Deleterious Endothelial-Adipocyte Cross-Talk in Patients With Heart Failure and Type 2 Diabetes

**DOI:** 10.1016/j.jacbts.2026.101527

**Published:** 2026-04-10

**Authors:** Oliver I. Brown, Katherine I. Bridge, Alexander-Francisco Bruns, Cheukyau Luk, Natallia Makava, Chew W. Cheng, Pradeep Wijayagoonawardana, Muzahir Tayebjee, Christopher E.D. Saunderson, Sam Straw, Michael Drozd, Anna Skroma, Natalie J. Haywood, Hema Viswambharan, Stephe Kamalathasan, John Gierula, David J. Beech, Khalid Naseem, Kathryn J. Griffin, Stephen B. Wheatcroft, Klaus K. Witte, Lee D. Roberts, Richard M. Cubbon, Mark T. Kearney

**Affiliations:** a Leeds Institute for Cardiovascular and Metabolic Medicine, University of Leeds, Leeds, United Kingdom; b Leeds Teaching Hospitals Trust, Leeds, United Kingdom

**Keywords:** cell cross-talk, endothelium, heart failure, senescence, type 2 diabetes mellitus

## Abstract

Subcutaneous adipose tissue microvascular endothelial cells (MVECs) from patients with type 2 diabetes mellitus and heart failure exhibit a senescent phenotype characterized by elevated senescence-associated secretory phenotype markers, reduced adenosine triphosphate production, and impaired angiogenic and proliferative capacities. When cocultured with healthy adipocytes, senescent MVECs drive adverse cross-talk, inducing a proinflammatory adipocyte phenotype with increased interleukin-6 expression and reduced glucose uptake. Digoxin reduces MVEC senescence and restores healthy cross-talk, improving glucose uptake and mitigating the effects of senescent MVECs on adipocytes.

## Highlights

• The contribution of endothelial senescence to SAT dysfunction in heart failure and type 2 diabetes in humans is unknown.
• We show that adipose endothelial cells from people with HFDM are senescent and in cross-talk with adipocytes results in a proinflammatory adipocyte phenotype resistant to glucose uptake.
• Moreover, we show that digoxin when used as a senolytic, reduces endothelial senescence, mitigating the negative cross-talk effects on adipocyte function.
• Future research will focus on the therapeutic potential of digoxin in vivo to ameliorate SAT dysfunction in HFDM patients.

## Visual Abstract

**Figure:**
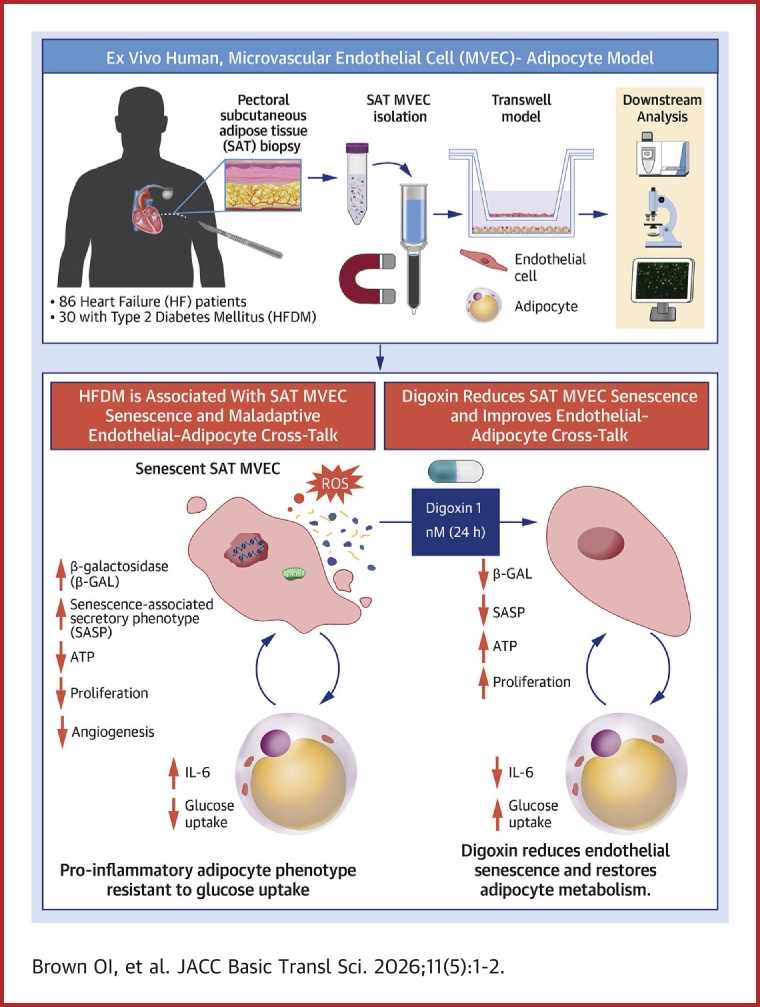

Heart failure (HF) with reduced ejection fraction and type 2 diabetes mellitus (T2DM) are global epidemics that frequently coexist.^1^, ^2^, ^3^ Despite recent advances in pharmacologic therapies, patients with both HF and T2DM (HFDM) have a prognosis worse than many malignancies.^4^^,^^5^ Subcutaneous adipose tissue (SAT) is a complex organ composed of a range of cell types, including white adipocytes, immune cells, stromal cells, and microvascular endothelial cells (MVECs).^6^^,^^7^ Normal SAT function is critical to metabolic homeostasis, and SAT function is dependent on a healthy microvasculature.^8^, ^9^, ^10^ This is thought to become dysregulated in T2DM, in which a vicious cycle of endothelial dysfunction, interstitial fibrosis, chronic inflammation, and impaired angiogenesis results in “SAT failure.”^11^^,^^12^

Senescence is the process of biological aging, characterized by cells’ undergoing stable cell-cycle arrest, leading to extensive changes in cell behavior and phenotype, including a reduction in mitochondrial function, reduced proliferative capacity, and oxidative stress.^13^ A key defining feature of senescence is an increase in the secretion of numerous inflammatory, extracellular matrix remodeling, and proliferative activity modulating factors known as the senescence-associated secretory phenotype (SASP).^14^ Obesity and chronic hyperinsulinemia, hallmarks of T2DM, are associated with an increase in premature adipocyte senescence, resulting in greater SASP expression and adipose tissue inflammation.^15^ However, the role of endothelial senescence contributing to SAT function is poorly understood.

Digoxin, a cardiac glycoside commonly used for atrial fibrillation, has been shown to have senolytic activity in chemotherapy-induced senescent murine models and cell lines.^16^ These studies, however, used supratherapeutic doses of digoxin,^16^ and the therapeutic potential on endothelial cells or primary human cells with disease has not been addressed.

In this study, we characterized the phenotype of SAT from patients with HFDM and, specifically, SAT microvascular endothelial cells (SATMVECs). We identified the presence and sequelae of SATMVEC senescence and its effects on human adipocyte function. Finally, we examined the role of digoxin in targeting SATMVEC senescence and SATMVEC-adipocyte cross-talk.

## Methods

### Patient recruitment and tissue collection

Patients with HF with or without T2DM undergoing elective cardiac implantable electronic device (CIED) insertion at the Leeds Teaching Hospitals Trust were recruited between 2018 and 2023. HF was defined as signs and symptoms of the disease with a documented left ventricular ejection fraction ≤40% on prior echocardiography or cardiac magnetic resonance imaging. Patients with significant valvular disease or restrictive, hypertrophic, or infiltrative cardiomyopathy were excluded. T2DM was a patient-reported diagnosis, but for inclusion, patients had to be taking antidiabetic medicine and/or have a serum glycated hemoglobin (HbA_1c_) concentration ≥48 mmol/mol. On the basis of these definitions, patients were classified as having HF or HFDM. The study was approved by the local ethics committee (11/YH/0291) and was conducted in accordance with the principals of the Declaration of Helsinki. All patients gave written informed consent prior to enrollment.

At the time of CIED insertion and under local anesthetic, a small (approximately 1-1.5 cm^3^ or 250-500 mg) piece of SAT was taken from the area superficial to pectoralis major. The tissue sample was immediately subdivided, with one piece being placed into MACS (Miltenyi Biotec) tissue storage solution (for endothelial cell extraction), one into 4% paraformaldehyde (for histologic analysis), and one into phosphate-buffered saline (for studies of angiogenesis and vascular density) and a final piece being flash frozen in liquid nitrogen (for RNA extraction and quantitative polymerase chain reaction). Blood samples were taken at the time of the procedure through the pacing lead sheath for routine biochemistry and hematology (including serum N-terminal pro–brain natriuretic peptide and HbA_1c_).

### Whole-SAT analysis

To evaluate structural and vascular characteristics of SAT, we performed histologic staining, quantified adipocyte size and collagen deposition, and assessed endothelial sprouting in ex vivo angiogenesis assays. Full methodological details are provided in the Supplemental Methods.^17^^,^^18^

### Endothelial cell isolation and culture

Human SATMVECs were isolated from samples of SAT as previously described.^19^ Experiments were performed in cells at passage 2 to passage 4. All cells demonstrated >95% purity on flow cytometry and maintained endothelial morphology until at least passage 5.

### Digoxin treatment of SATMVECs

For digoxin-treated SATMVEC assays, fresh microvascular endothelial cell growth medium containing 1 nM digoxin was prepared by serial dilution of a 100 mM stock solution of digoxin prepared in dimethyl sulfoxide and was applied to cells for 24 hours. Medium containing only dimethyl sulfoxide was used as a control. The concentration of 1 nM digoxin was chosen as it falls within the therapeutic range used clinically in humans (0.5-2 ng/mL) and did not demonstrate toxicity to SATMVECs during cell viability assays (LIVE/DEAD, Thermo Fisher Scientific).

### Assessment of endothelial cell phenotype

Full methodological details are provided in the Supplemental Appendix; a concise overview follows.

### RNA sequencing

RNA was extracted from SATMVECs at passage 2 using TRIzol reagent (Invitrogen), and concentration and purity were assessed using NanoDrop (Thermo Fisher Scientific) spectrophotometry. RNA sequencing libraries were prepared from 600 ng total RNA per sample and sequenced by Novogene. Raw reads were quality trimmed and aligned to the human reference genome (GRCh38.p14) using STAR (Spliced Transcripts Alignment to a Reference) aligner, and gene counts were generated using FeatureCounts. Differentially expressed genes were identified using DESeq2, with batch correction applied using the sva package to account for sequencing date.^20^ Hypothesis-driven analysis was performed focusing on canonical senescence genes (p16, p21, p53, and RB1), and Bonferroni correction was applied for multiple testing. Further methodological details are provided in the Supplemental Methods.

### Endothelial phenotyping assays

SATMVEC functional characteristics were evaluated across proliferation, tube formation, mitochondrial function, and senescence markers. Cell proliferation was quantified using the Click-iT EdU incorporation assay (Thermo Fisher Scientific), and angiogenic potential was assessed using tube formation assays on Matrigel (Corning). Cellular senescence was measured by senescence-associated beta-galactosidase (SA-β-gal) activity using the CellEvent Senescence Green Detection Kit (Thermo Fisher Scientific). Mitochondrial respiration were determined using the Seahorse XF Cell Mito Stress Test (Agilent Technologies). Image analysis was conducted in ImageJ (National Institutes of Health) by investigators (O.I.B. and K.I.B.) blinded to study group. Detailed plating densities, assay timings, and reagent concentrations are described in the Supplemental Methods.^19^

### Endothelial secretome profiling

To characterize the SASP, conditioned media from confluent SATMVEC monolayers were collected after 24-hour incubation in unsupplemented medium. Cytokine quantification was performed using the MAGPIX Human Cytokine 10-Plex panel (Thermo Fisher Scientific), with results normalized to total protein content. Only replicate pairs with coefficients of variation <10% were included in analyses.

Not all primary SATMVEC isolations yielded sufficient viable cells to perform every downstream assay. For most donors, approximately 4 or 5 cryovials per sample were obtained (frozen at passage 2), each containing about 250,000 endothelial cells. These cells could typically be expanded only to passage 4 following reanimation. In some instances, cell yield or post-thaw viability was insufficient for all experimental assays, particularly those requiring greater cell numbers. Consequently, the number of biological replicates varied among assays and is indicated in the corresponding figure legends.^21^

### Adipocyte culture, coculture setup, and phenotyping

Human subcutaneous white preadipocytes (Lonza, passage 2) from a single healthy donor were cultured and differentiated into mature adipocytes using standard protocols, as previously described.^19^ Once fully differentiated, adipocytes were cocultured for 24 hours with SATMVECs (passage 4) grown to confluence on 0.4-μm Transwell inserts (Corning) in microvascular endothelial cell growth medium (PromoCell). Adipocyte RNA, protein, and glucose uptake were subsequently assessed.

Glucose uptake was quantified using 2-NBD-glucose (20 μM, 30-minute incubation), followed by fixation and fluorescent imaging (ZOOM, IncuCyte). Quantification was performed using ImageJ, and analyses were independently verified by 2 blinded investigators (O.I.B. and K.I.B.).

To examine GLUT4 localization, cell-surface biotinylation was performed after 24 hours of coculture. Plasma-membrane proteins were labeled with EZ-Link Sulfo-NHS-SS-Biotin (Thermo Fisher Scientific), isolated using magnetic streptavidin beads, and analyzed using western blotting for GLUT4, with HSP90 used as a loading control.

For digoxin-treated experiments, confluent SATMVECs were preincubated for 24 hours in 1 nM digoxin or vehicle control prior to coculture. Following coculture, adipocyte functional assays were performed as described earlier.

Full methodological details are provided in the Supplemental Methods.

### Statistical methods

Continuous data are presented as mean ± SD for baseline characteristics and as mean ± SEM for experimental data. Categorical data are presented as counts with percentages. Normality was assessed using the Shapiro-Wilk test. Differences between the HF and HFDM groups were assessed using either Student’s *t*-test or the Mann-Whitney *U* test (if not normally distributed) for continuous variables and the chi-square test for categorical variables.

For experiments assessing the effects of digoxin, data are expressed as fold change relative to paired control conditions to account for high interindividual variability and highlight within-donor responses. This normalization strategy was chosen a priori to maximize statistical power in a small cohort and reduce the influence of baseline interdonor variability. For SATMVEC assays, each patient’s digoxin-treated value was standardized to their own untreated control result. For adipocyte assays, there was inherently less biological variability because all cells were derived from a single genetic donor; therefore, data were standardized to the mean HF control value for each assay.

Differences between digoxin and control within each HF and HFDM group category were assessed using paired Student’s *t*-tests or Wilcoxon signed rank tests, while associations between 2 continuous variables was assessed using Pearson’s correlation coefficient (*r*). Scatterplots were produced, and linear regression models were constructed for SA-β-gal and clinical characteristics adjusting for age and biological sex, with results presented as the regression coefficient with its 95% CI. All statistical tests were 2-sided, and statistical significance was defined as *P* < 0.05. Missing data were not imputed. We did not account for multiple testing between experimental methods, as these addressed the same hypothesis with complementary approaches, meaning that post hoc adjustments are inappropriate.

Analyses were performed using Stata/MP version 18 (StataCorp), R version 4.1.1 (R Core Team), or Prism version 9.0 (GraphPad Software).

## Results

We took biopsies of pectoral SAT from 86 patients with HF with or without T2DM undergoing insertion of CIEDs. There were no statistically significant differences in baseline demographic or clinical characteristics between groups, including age, body mass index, disease severity, comorbidities, serum biochemistry, and medication use (aside from hydroxymethylglutaryl coenzyme A reductase inhibitors and antidiabetic medications). No patients were taking digoxin at enrollment (Table 1).

**Table 1:** Population Characteristics of Subcutaneous Adipose Tissue Biopsy Study Participants

	HF (n = 56)	HFDM (n = 30)	*P* Value
Sociodemographic			
Age, y	70 ± 13	69 ± 11	0.90
Male	32 (57.1)	23 (76.7)	0.045
Ever smoked	12 (21.4)	9 (30.0)	0.38
BMI, kg/m^2^	29.5 ± 5.3	31.9 ± 6.9	0.080
Disease severity			
Ischemic etiology	26 (46.4)	14 (46.7)	0.98
LVEF, %	30 ± 10	29 ± 10	0.68
Diabetes duration, y	—	13.3 ± 12.5	—
HbA_1c_, mmol/mol	40 ± 3	57 ± 14	<0.001
NT-proBNP, pg/mL	3,355 ± 1,049	4,595 ± 1,473	0.48
NYHA functional class ≥ III	13 (23.2)	11 (36.7)	0.19
Comorbidities			
Atrial fibrillation	15 (26.7)	13 (43.3)	0.13
COPD	2 (3.6)	3 (10.0)	0.23
HTN	9 (16.1)	8 (26.7)	0.26
Blood results			
Hb, g/L	138 ± 15	137 ± 20	0.66
Na, mmol/L	139 ± 3	139 ± 4	0.71
K, mmol/L	4.4 ± 0.4	4.7 ± 0.5	0.016
eGFR, mL/min/1.73 m^2^	63 ± 19	67 ± 20	0.32
HOMA-IR	1.4 ± 1.1	2.6 ± 2.3	0.062
Interleukin-6, pg/mL	3.9 ± 2.5	8.1 ± 6.2	0.003
Interleukin-8, pg/mL	7.0 ± 5.9	7.3 ± 5.2	0.80
Medications			
ACEI/ARB/ARNI	48 (85.7)	26 (86.7)	0.93
Beta-blocker	49 (87.5)	26 (86.7)	0.90
MRA	30 (53.5)	16 (53.3)	0.92
Aspirin	15 (26.8)	8 (26.7)	0.91
Statin	31 (55.4)	24 (80.0)	0.029
SGLT2 inhibitor	9 (16.1)	8 (26.7)	0.26
Metformin	0 (0.0)	18 (60.0)	<0.001
Insulin	0 (0.0)	5 (16.7)	<0.001

Values are mean ± SD or n (%). *P* values were calculated using Student’s *t*-test or the chi-square test for continuous and categorical data, respectively, between the HF and HFDM groups.

ACEI = angiotensin-converting enzyme inhibitor; ARB = angiotensin receptor blocker; ARNI = angiotensin receptor neprilysin inhibitor; BMI = body mass index; COPD = chronic obstructive pulmonary disease; eGFR = estimated glomerular filtration rate; HbA_1c_ = glycated hemoglobin; Hb = hemoglobin; HF = heart failure; HFDM = heart failure and type 2 diabetes mellitus; HOMA-IR = homeostatic model assessment of insulin resistance; HTN = hypertension; LVEF = left ventricular ejection fraction; MRA = mineralocorticoid receptor antagonist; NT-proBNP = N-terminal pro–brain natriuretic peptide; SGLT2 = sodium-glucose cotransporter 2.

First, we examined the effects of T2DM on whole-SAT phenotype. SAT from patients with HFDM had hypertrophied white adipocytes, increased collagen deposition, impaired microvascular expansion with fewer blood vessels, and impaired angiogenesis. (Supplemental Figures 1A to 1F).

Next, we isolated SATMVECs to characterize functional cellular behaviors and their contribution to impaired SAT microvascular expansion. We found that there was no difference in basal SATMVEC protein expression of insulin-like growth factor-1 or insulin receptor, but expression of endothelial nitric oxide synthase was lower among patients with HFDM (Supplemental Figures 2A to 2C).

**Figure 1:**
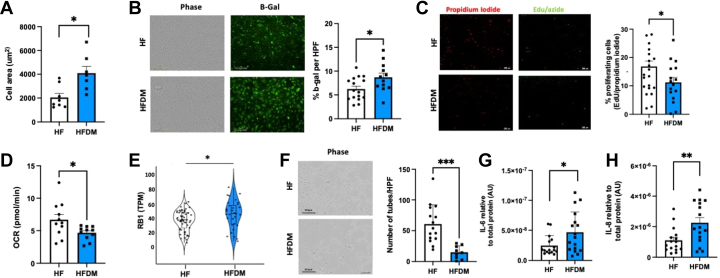
SATMVECs From Patients With HFDM Exhibit a Senescent Phenotype (A) Subcutaneous adipose tissue microvascular endothelial cell (SATMVEC) size (n = 8, 7). (B) SATMVEC senescence histochemical senescence-associated β-galactosidase (b-gal) staining with illustrative phase and fluorescent imaging (n = 16, 12). (C) SATMVEC proliferation using the Click-iT EdU imaging kit (n = 19, 14). (D) SATMVEC mitochondrial adenosine triphosphate production (n = 12, 11). (E) Messenger RNA expression of RB1 in SATMVEC plotted with jittered data points. Significance is Bonferroni adjusted (n = 56, 30). (F) SATMVEC tube forming in Matrigel with illustrative phase images (n = 15, 9). (G) Secreted interleukin (IL)–6 in SATMVEC conditioned medium standardized to total protein concentration quantified using Luminex (Sigma-Aldrich) (n = 16, 17). (H) Secreted IL-8 in SATMVEC conditioned medium standardized to total protein concentration quantified using Luminex (n = 16, 16). Data are presented as mean ± SEM, with individual data points representing biological replicates, unless otherwise indicated. Violin plots show the distribution of individual values. *P* values for comparisons between the heart failure (HF) and HF with type 2 diabetes mellitus (HFDM) groups were calculated using Student’s *t*-test or the Mann-Whitney *U* test as appropriate according to data distribution. ∗*P* < 0.05, ∗∗*P* < 0.01, and ∗∗∗*P* < 0.001. AU = arbitrary units; HPF = high power field; OCR = oxygen consumption rate; TPM = transcripts per million.

**Figure 2:**
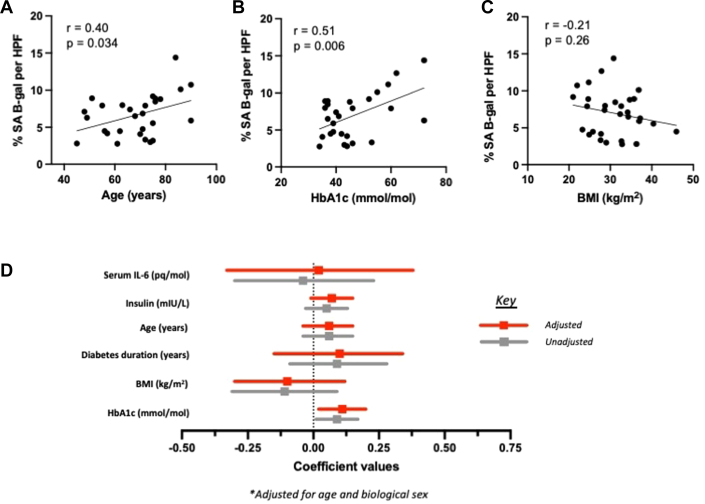
Association of Endothelial SA B-gal With Clinical Characteristics (A) Scatterplot of senescence-associated beta-galactosidase (SA B-gal) vs age with Pearson’s correlation coefficient (n = 27). (B) Scatterplot of SA B-gal vs glycated hemoglobin (HbA_1c_) with Pearson’s correlation coefficient (n = 27). (C) Scatterplot of SA B-gal vs body mass index (BMI) with Pearson’s correlation coefficient (n = 27). (D) Unadjusted and adjusted linear regression of SA B-gal by clinical characteristics. Abbreviations as in Figure 1.

Compared with those from patients with HF alone, SATMVECs from patients with HFDM were phenotypically senescent, evidenced by increased cell area, increased expression of SA-β-gal (a histochemical marker of senescence) (Figures 1A and 1B), reduced proliferative capacity (Figure 1C), reduced mitochondrial-generated adenosine triphosphate (Figure 1D), and increased superoxide production (Supplemental Figure 2E). There were no significant differences in other mitochondrial functions, including basal and maximal respiration and spare respiratory capacity (Supplemental Figures 2F to 2H). RNA sequencing revealed a significant increase in the expression of RB1 messenger RNA (mRNA), a pivotal inhibitor of cell cycle–promoting genes, in SATMVECs from patients with HFDM (Figure 1E), which was validated using reverse transcriptase quantitative polymerase chain reaction (Supplemental Figure 2I). However, there was no significant difference in expression in other canonical regulators of the cell cycle (Supplemental Figures 2J to 2L). SA-β-gal expression within SATMVECs was positively correlated with serum HbA_1c_, with HbA_1c_ the only age- and sex-adjusted positive correlate of SA-β-gal (Figures 2A to 2D), demonstrating that T2DM severity was likely responsible for the senescent phenotype of SATMVECs. SATMVEC in vitro tube formation isolated from patients with HFDM was impaired compared with SATMVECs from patients with HF alone (Figure 1F). Analysis of the secretome of SATMVECs demonstrated evidence of a SASP among patients with HFDM, with higher concentrations of interleukin (IL)–6 and IL-8 compared with SATMVECs from patients with HF alone (Figures 1G and 1H).

To ensure that SATMVECs from patients with HF alone did not exhibit increased senescence compared with those without HF, we performed detailed phenotyping analyses of SATMVECs from a control group of patients (n = 25) undergoing CIED implantation without HF or HFDM (baseline characteristics are provided in Supplemental Table 1). We observed no differences in cell size, SA-β-gal expression, proliferative capacity, or RB1 expression between SATMVECs from patients with HF and those from control subjects (Supplemental Figures 3A to 3D). These findings indicate that SATMVECs from patients with HF are not overtly senescent and therefore serve as an appropriate comparator group for those from patients with HFDM.

**Figure 3:**
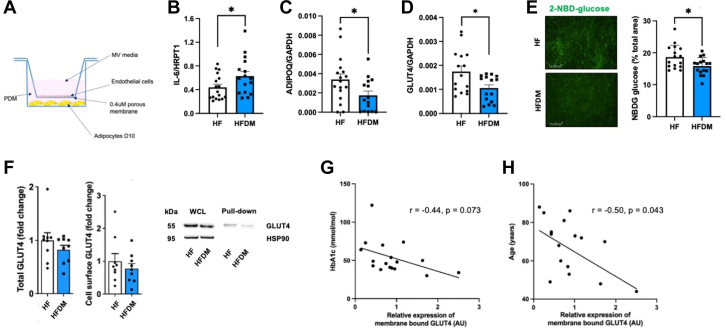
Cross-Talk Between HFDM SATMVECs and Adipocytes Induces a Proinflammatory, Metabolically Dysregulated Phenotype (A) Illustrative diagram showing set up of coculture experiments between SATMVECs and adipocytes. (B) Expression of adipocyte IL-6 messenger RNA (mRNA) following 24-hour coculture with SATMVECs (n = 17, 16). (C) Expression of adipocyte ADIPOQ mRNA following 24-hour coculture with SATMVECs (n = 17, 15). (D) Expression of adipocyte GLUT4 mRNA following 24-hour coculture with SATMVECs (n = 15, 15). (E) Uptake of 2-NBD-glucose in adipocytes cocultured with SATMVECs for 24 hours with illustrative images (n = 16, 16). (F) Quantification and representative immunoblot of total and cell-surface GLUT4 protein in adipocytes cocultured with SATMVECs from HF and HFDM patients for 24 hours (n = 9, 10). (G) Correlation between relative membrane-bound GLUT4 expression in adipocytes and patient HbA_1c_ levels. (H) Correlation between relative membrane-bound GLUT4 expression in adipocytes and patient age. Data are presented as mean ± SEM, with individual data points representing biological replicates. *P* values for comparisons between the HF and HFDM groups were calculated using Student’s *t*-test or the Mann-Whitney *U* test as appropriate on the basis of data distribution. *P* values for correlation analyses in G and H were calculated using Pearson’s correlation coefficient. ∗*P* < 0.05. GAPDH = glyceraldehyde-3-phosphate dehydrogenase; MV = microvascular; PDM = preadipocyte differentiation media; WCL = whole-cell lysate; other abbreviations as in Figures 1 and 2.

Next, we established a coculture system to investigate SATMVEC-adipocyte cross-talk (Figure 3A).^16^ Following coculture with SATMVECs from patients with HFDM, adipocytes from a healthy donor (commercially purchased) demonstrated an inflammatory phenotype with significantly increased IL-6 and reduced adiponectin (ADIPOQ) mRNA expression (Figures 3B and 3C). Moreover, adipocytes cocultured with SATMVECs from patients with HFDM had reduced GLUT4 mRNA expression and lower uptake of 2-NBD-glucose, illustrating a clear impact on adipocyte health and function as a result of SATMVEC dysfunction (Figures 3D and 3E). To further assess whether these transcriptional changes were accompanied by alterations in GLUT4 protein or trafficking, we performed GLUT4 translocation assays to examine the effects of SATMVEC coculture on adipocyte glucose homeostasis. We observed no significant differences in total or surface-bound GLUT4 protein between adipocytes cocultured with SATMVECs from individuals with HF or HFDM (Figure 3F). However, surface-bound GLUT4 showed a negative correlation with serum HbA_1c_ and patient age (Figures 3G and 3H), 2 clinical parameters that were significantly correlated with SATMVEC SA-β-gal expression.

**Figure 4:**
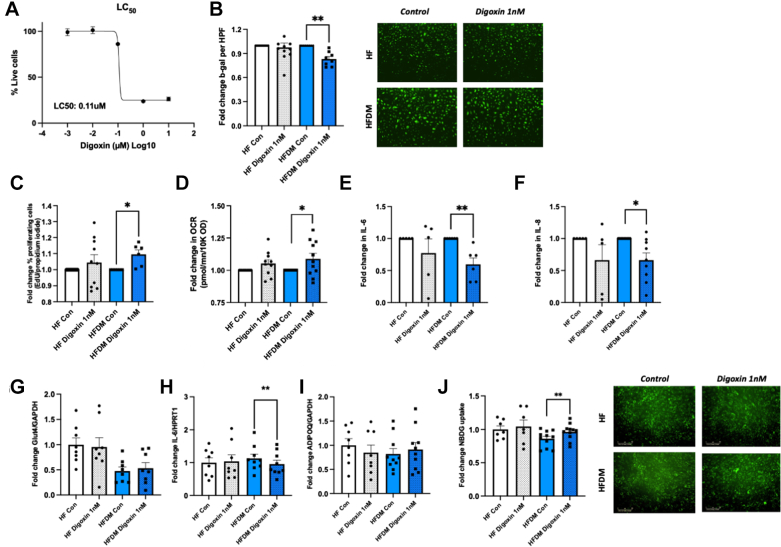
Digoxin Reduces SATMVEC Senescence and Restores Healthy Adipocyte Glucose Homeostasis (A) Median concentration curve (50% lethal concentration [LC_50_]) of digoxin on human SATMVECs using LIVE/DEAD assay (n = 5). (B) Fold change in SATMVEC expression of senescence-associated β-galactosidase after culture in 1 nM digoxin vs control medium for 24 hours, with illustrative fluorescein stain images (n =11, 11, 8, 8). (C) Fold change in SATMVEC proliferation measured using the Click-iT EdU imaging kit after culture in 1 nM digoxin vs control medium for 24 hours (n =10, 10, 6, 6). (D) Fold change in SATMVEC mitochondrial adenosine triphosphate production measured using the Mito Stress Test after culture in 1 nM digoxin vs control medium for 24 hours (n = 10, 11). (E,F) Fold change in SATMVEC-secreted IL-6 (n = 5, 5, 7, 7) (E) and IL-8 (F) expression into conditioned medium following culture in 1 nM digoxin vs control medium for 24 hours (n = 5, 5, 9, 9. (G) Adipocyte GLUT4 mRNA expression following coculture with SATMVECs treated with 1 nM digoxin vs control medium for 24 hours, standardized to HF control (n = 8, 8, 8, 8). (H) Adipocyte IL-6 mRNA expression following coculture with SATMVECs treated with 1 nM digoxin vs control medium for 24 hours, standardized to HF control (n = 8, 8, 9, 9). (I) Adipocyte ADIPOQ mRNA expression following coculture with SATMVECs treated with 1 nM digoxin vs control medium for 24 hours, standardized to HF control (n = 8, 8, 10, 10). Data are presented as mean ± SEM, with individual data points representing biological replicates. *P* values for paired comparisons between control and digoxin-treated samples were calculated using paired Student’s *t*-test or Wilcoxon signed rank test as appropriate according to data distribution. (J) Fold change in 2-NBD-glucose uptake assay with adipocytes cocultured with SATMVECs treated with 1 nM digoxin vs control medium for 24 hours, with illustrative uuorescein stain images (n = 7, 7, 11, 11). ∗*P* < 0.05 and ∗∗*P* < 0.01. Con = control; other abbreviations as in Figure 1, Figure 2, Figure 3.

To investigate the potential senolytic role of digoxin on SATMVECs, we cultured SATMVECs in digoxin-containing medium for 24 hours. Digoxin demonstrated no cellular toxicity within the therapeutic range (Figure 4A) and had a median lethal toxicity at 0.11 μM (equivalent to 85.8 ng/μL). We therefore used a digoxin concentration of 1 nM for all subsequent experiments, as this was equivalent to approximately 0.8 ng/μL (within the therapeutic range for serum digoxin concentration). Treatment of SATMVECs from patients with HFDM with digoxin reduced SA-β-gal expression and increased adenosine triphosphate production and proliferative capacity (Figures 4B to 4D). IL-6 and IL-8 expression was reduced in the conditioned medium of SATMVECs from patients with both HF and HFDM following 24 hours of culture in 1 nM digoxin (Figures 4E and 4F).

Having demonstrated that digoxin reduced SATMVEC senescence, we analyzed the effects of digoxin-treated SATMVECs on adipocytes. Although adipocyte mRNA expression of ADIPOQ was not significantly different, IL-6 mRNA expression was significant reduced following coculture with digoxin-treated SATMVECs from patients with HFDM (Figures 4G to 4I). Moreover, we observed an increase in 2-NBD-glucose uptake in adipocytes cocultured with digoxin-treated SATMVECs (Figure 4J), demonstrating an improved immunometabolic adipocyte phenotype.

## Discussion

Our study provides a detailed analysis and novel insights into the role of SATMVECs in the pathophysiology of HFDM. By characterizing the senescent phenotype of SATMVECs in HFDM, we demonstrate their contribution to impaired microvascular function and subsequent adipocyte dysfunction. For the first time in humans, we show that digoxin, a widely used cardiac glycoside, mitigates SATMVEC senescence and adverse SATMVEC-adipocyte cross-talk. This results in an improved adipocyte metabolic and inflammatory phenotype, highlighting a potential therapeutic avenue for addressing adipose tissue dysfunction in this high-risk population.

Senescent cells are thought to induce an inflammatory state through the actions of the SASP, which provokes local tissue damage leading to a persistent, self-reinforcing, inflammatory microenvironment.^22^^,^^23^ A key constituent of the SASP is IL-6, and we observed significantly increased levels of IL-6 in the endothelial secretome from patients with HFDM compared with HF alone. Serum IL-6 was also significantly increased in patients with HFDM. Although we were unable to identify the systemic source of IL-6 in our study population, the major producer of IL-6 in vivo is SAT.^24^^,^^25^ Prior evidence demonstrating deleterious cell-cell communication facilitated by the SASP has been generated using animal models. Barinda et al^26^ demonstrated that genetically induced endothelial cell–specific senescence in mice leads to adipocyte and whole-body insulin resistance. However, no study has demonstrated SATMVEC senescence in humans with T2DM or its effect on adipocyte function.

Senescence is a complex cellular process, and quantification of senescence both in vitro and in vivo remains challenging,^27^ particularly as markers of cellular senescence can differ among cell lineages, tissue beds, and organisms.^28^ We therefore used a multifaceted approach to show definitive evidence of increased cellular senescence, including morphologic, histochemical, genetic, and functional differences, as defined in recent guidelines.^29^

The age profile of our cohort reflects a typical HF population and may contribute to the senescent phenotype observed. Aging is associated with endothelial cell senescence, driven by impaired mitochondrial biogenesis, reduced angiogenic capacity, vascular inflammation, and arterial stiffness.^30^ It also promotes loss of regenerative potential and increased expression of canonical senescence markers across multiple tissues, including the vasculature.^31^ Although age did not differ between groups, this background provides a clinically relevant context and supports the concept that SATMVEC senescence may represent a mechanistic link among aging, diabetes, and microvascular dysfunction in this cohort.

Our data raise the intriguing possibility that digoxin may be used as a senolytic agent to repair dysfunctional SAT in patients with T2DM. Previous studies investigating the senolytic actions of digoxin did not explore its role in SATMVEC-related senescence and used supratherapeutic doses of digoxin that would be toxic if used in humans,^16^^,^^32^ whereas ours is the first study to demonstrate the senolytic activity of digoxin at safe therapeutic dose in humans. It is well established that digoxin safely improves hemodynamic status, alleviates symptoms, and improves the deleterious neurohumoral profile of patients with advanced HF.^33^ In the DIG (Digitalis Investigation Group) trial, digoxin safely reduced the risk for HF death and hospitalization among patients with chronic HF and left ventricular ejection fractions <45%.^34^ However, in that trial, diabetes was a self-declared diagnosis, limited biochemical investigations were reported, and HbA_1c_ and fasting or nonfasting glucose was not included. In addition, although digoxin toxicity was more prevalent among patients with diabetes than without (6.5% vs 5.8%), subgroup analysis of the DIG trial supports the safety of digoxin in patients with HF and clinical diagnoses of diabetes per se.^35^ Despite these data, digoxin use has fallen substantially over the past 2 decades; once prescribed to 80% of patients with HF, it is now taken by fewer than 40%.^36^ The ongoing DECISION (Digoxin Evaluation in Chronic Heart Failure: Investigational Study in Outpatients in the Netherlands) trial, a large double-blind, placebo-controlled trial of low-dose digoxin at serum concentrations between 0.5 and 0.9ng/mL (a concentration used in our present study in vitro) or placebo in patients with HF and left ventricular ejection fractions of <50%, may provide further supportive data for the use of digoxin among patients with HF and diabetes and address concerns about toxicity.^37^ Moreover, the DECISION trial will also include a biomarker substudy that may provide further mechanistic insight to support the intriguing possibility that the senolytic actions of digoxin demonstrated in our study could potentially normalize SAT dysfunction in patients with advanced HFDM, thereby improving outcomes in these highest risk patients.

Our findings raise important translational questions regarding how the senolytic properties of digoxin might be harnessed clinically to improve outcomes in patients with HFDM. However, the potential repurposing of digoxin as a senolytic therapy is not without challenges. Its narrow therapeutic window, risk for toxicity, and variable pharmacokinetics limit its widespread use, particularly in patients with renal impairment or polypharmacy, features common in HFDM. Furthermore, a validated clinical readout of senolytic efficacy is lacking, making it difficult to directly assess in vivo benefit. Future studies will therefore need to determine whether digoxin, at contemporary therapeutic concentrations, exerts measurable senolytic effects in humans and to define the specific patient populations in which its risk-benefit profile would support such use.

Several other pharmacologic agents commonly used in the treatment of patients with T2DM have been shown to exert pleiotropic effects on cellular metabolism and aging pathways that could influence endothelial phenotype. Metformin, a first-line therapy for T2DM, has previously been reported to confer geroprotective and antisenescent effects in human cell lines and nonhuman primates,^38^^,^^39^ with ongoing clinical trials investigating its broader role in delaying age-related decline.^40^ Although the senolytic actions of metformin in endothelial cells are not fully understood, we were still able to observe a clear senescent phenotype in SATMVECs derived from patients with HFDM, despite 60% of the cohort receiving metformin, and these differences persisted after adjustment for HbA_1c_. Sodium-glucose cotransporter 2 inhibitors have been shown to reduce endothelial senescence and inflammation in vitro and to decrease adipose tissue senescent load and metabolic dysfunction in vivo.^41^, ^42^, ^43^, ^44^ Thus, these data collectively suggest that contemporary antidiabetic therapies may modulate vascular aging and adipose-endothelial cross-talk and should be considered in future mechanistic studies investigating microvascular dysfunction in HFDM.

### Study limitations

Our study has many strengths, including SAT microvasculature phenotyping on a histologic and cellular level, the use of samples collected from patients, and our sample size. However, because of the limited yield of SATMVECs from tissue isolation and the limited ability of SATMVECs to undergo serial passages, we were unable to repeat all experiments on every sample. Moreover, we were unable to biopsy other SAT depots in which microvascular function may differ. A control cohort without HF or T2DM was included to provide baseline SATMVEC phenotyping data. However, the HF group remained the most appropriate primary comparator for HFDM, as it helped ensure the standardization of existing pharmacologic therapies across the patient groups, ensuring that, except for T2DM, the 2 cohorts were as well matched as possible. Importantly, our findings indicate that the coexistence of T2DM and HF exerts a synergistic and distinctive effect on SATMVEC senescence, rather than representing a simple additive consequence of either condition alone.

## Conclusions

We have demonstrated that T2DM induces SATMVEC senescence in patients with HF, which, through the secretion of inflammatory mediators, may directly affect adipocyte function. Moreover, we have shown for the first time that digoxin, used in vitro at a therapeutic dose, reduces SATMVEC senescence, remediates healthy SATMVEC-adipocyte cross-talk, and promotes a healthy adipocyte phenotype.

### Data availability

The data sets generated in the present study may be available from the corresponding author upon reasonable request.

Perspectives **COMPETENCY IN MEDICAL KNOWLEDGE:** This study identifies in vitro that endothelial cell senescence within SAT is a mechanistic contributor to adipose dysfunction in patients with HFDM. Senescent adipose MVECs promote maladaptive endothelial-adipocyte cross-talk, characterized by inflammatory activation and impaired adipocyte glucose uptake. These findings expand the current understanding of cardiometabolic disease by highlighting endothelial senescence as a biologically relevant and potentially modifiable pathway linking diabetes, HF, and adipose tissue dysfunction.

**COMPETENCY IN PATIENT CARE AND PROCEDURAL SKILLS:** Recognition of adipose tissue microvascular health as a determinant of systemic metabolic homeostasis underscores the importance of integrated cardiovascular-metabolic care in patients with HFDM. The observation that therapeutic-range digoxin attenuates endothelial senescence and improves downstream adipocyte metabolic function provides a mechanistic rationale for targeting senescent endothelial cells as a novel strategy to restore adipose tissue health. Our work supports the need for rigorous in vivo studies to establish physiological relevance, safety, and efficacy before consideration of clinical translation.

**TRANSLATIONAL OUTLOOK:** Future translational studies should determine whether digoxin exerts senolytic effects on adipose tissue microvasculature in vivo and whether these effects translate into meaningful improvements in metabolic control and clinical outcomes in patients with HFDM. Prospective clinical trials incorporating biomarkers of endothelial senescence and adipose tissue function will be essential to define the therapeutic potential, safety profile, and patient populations most likely to benefit from senescence-targeted interventions.

## Funding Support and Author Disclosures

Dr Kearney holds a British Heart Foundation Chair in Cardiovascular and Diabetes Research, which also funded Dr Brown and Ms Makava (CH/13/1/30086). Drs Brown, Drozd, and Straw are supported by the National Institute for Health and Care Research. This research is supported by the National Institute for Health and Care Research Leeds Biomedical Research Centre (NIHR203331). The Faculty of Biological Sciences, University of Leeds, Bioimaging Facility, has received equipment grants from the Wellcome Trust to purchase the confocal microscopes used in this project. Dr Drozd was funded by a British Heart Foundation Clinical Research Training Fellowship (FS/18/44/33792). Dr Roberts was funded by a Diabetes UK RD Lawrence Fellowship (16/0005382), the Biotechnology and Biological Sciences Research Council (BB/R013500/1), and the Medical Research Council (MR/X009734/1). Dr Straw was funded by a British Heart Foundation Clinical Research Training Fellowship (FS/CRTF/20/24071). Dr Cubbon was funded by British Heart Foundation Clinical Intermediate Fellowships (FS/12/80/29821). Dr Bruns and Ms Skroma were funded by a British Heart Foundation Programme grant (RG/15/7/31521). Dr Brown has received speaker fees and honoraria from Novartis. Dr Straw has received speaker fees, honoraria, and nonfinancial support from AstraZeneca. Dr Cubbon has received speaker fees from Janssen Oncology. Dr Kearney has received honoraria from AstraZeneca; has received speaker fees from Merck and Novo Nordisk; and has received unrestricted research awards from AstraZeneca and Medtronic. All other authors have reported that they have no relationships relevant to the contents of this paper to disclose.

## References

[1] Ziaeian B., Fonarow G.C. (2016). Epidemiology and aetiology of heart failure. Nat Rev Cardiol.

[2] Guariguata L., Whiting D.R., Hambleton I. (2014). Global estimates of diabetes prevalence for 2013 and projections for 2035. Diabetes Res Clin Pract.

[3] Brown O.I., Drozd M., McGowan H. (2023). Relationship among diabetes, obesity, and cardiovascular disease phenotypes: a UK Biobank cohort study. Diabetes Care.

[4] Mamas M.A., Sperrin M., Watson M.C. (2017). Do patients have worse outcomes in heart failure than in cancer? A primary care-based cohort study with 10-year follow-up in Scotland. Eur J Heart Fail.

[5] Boden G., Homko C., Barrero C.A. (2015). Excessive caloric intake acutely causes oxidative stress, GLUT4 carbonylation, and insulin resistance in healthy men. Sci Transl Med.

[6] Rosen E.D., Spiegelman B.M. (2014). What we talk about when we talk about fat. Cell.

[7] Gustafson B., Hedjazifar S., Gogg S. (2015). Insulin resistance and impaired adipogenesis. Trends Endocrinol Metab.

[8] Hammarstedt A., Gogg S., Hedjazifar S. (2018). Impaired adipogenesis and dysfunctional adipose tissue in human hypertrophic obesity. Physiol Rev.

[9] Cao Y. (2007). Angiogenesis modulates adipogenesis and obesity. J Clin Invest.

[10] Norreen-Thorsen M., Struck E.C., Öling S. (2022). A human adipose tissue cell-type transcriptome atlas. Cell Rep.

[11] Crewe C., An Y.A., Scherer P.E. (2017). The ominous triad of adipose tissue dysfunction: inflammation, fibrosis, and impaired angiogenesis. J Clin Invest.

[12] Brown O.I., Bridge K.I., Kearney M.T. (2021). Nicotinamide adenine dinucleotide phosphate oxidases in glucose homeostasis and diabetes-related endothelial cell dysfunction. Cells.

[13] Di Micco R., Krizhanovsky V., Baker D., d’Adda di Fagagna F. (2021). Cellular senescence in ageing: from mechanisms to therapeutic opportunities. Nat Rev Mol Cell Biol.

[14] Zhang L., Pitcher L.E., Yousefzadeh M.J. (2022). Cellular senescence: a key therapeutic target in aging and diseases. J Clin Invest.

[15] Li Q., Hagberg C.E., Silva Cascales H. (2021). Obesity and hyperinsulinemia drive adipocytes to activate a cell cycle program and senesce. Nat Med.

[16] Triana-Martínez F., Picallos-Rabina P., Da Silva-Álvarez S. (2019). Identification and characterization of cardiac glycosides as senolytic compounds. Nat Commun.

[17] Junqueira L.C., Bignolas G., Brentani R.R. (1979). Picrosirius staining plus polarization microscopy, a specific method for collagen detection in tissue sections. Histochem J.

[18] Luk C., Bridge K.I., Warmke N. (2025). Paracrine role of endothelial IGF-1 receptor in depot-specific adipose tissue adaptation in male mice. Nat Commun.

[19] Brown O.I., Bridge K.I., Straw S. (2024). Studying adipose endothelial cell/adipocyte cross-talk in human subcutaneous adipose tissue. J Vis Exp.

[20] Love M.I., Huber W., Anders S. (2014). Moderated estimation of fold change and dispersion for RNA-seq data with DESeq2. Genome Biol.

[21] Viswambharan H., Yuldasheva N.Y., Imrie H. (2021). Novel paracrine action of endothelium enhances glucose uptake in muscle and fat. Circ Res.

[22] He S., Sharpless N.E. (2017). Senescence in health and disease. Cell.

[23] Khosla S., Farr J.N., Tchkonia T., Kirkland J.L. (2020). The role of cellular senescence in ageing and endocrine disease. Nat Rev Endocrinol.

[24] Mohamed-Ali V., Goodrick S., Rawesh A. (1997). Subcutaneous adipose tissue releases interleukin-6, but not tumor necrosis factor-alpha, in vivo. J Clin Endocrinol Metab.

[25] Mohamed-Ali V., Goodrick S., Bulmer K. (1999). Production of soluble tumor necrosis factor receptors by human subcutaneous adipose tissue in vivo. Am J Physiol.

[26] Barinda A.J., Ikeda K., Nugroho D.B. (2020). Endothelial progeria induces adipose tissue senescence and impairs insulin sensitivity through senescence associated secretory phenotype. Nat Commun.

[27] Gil J. (2023). The challenge of identifying senescent cells. Nat Cell Biol.

[28] González-Gualda E., Baker A.G., Fruk L., Muñoz-Espín D. (2021). A guide to assessing cellular senescence in vitro and in vivo. FEBS J.

[29] Ogrodnik M., Carlos Acosta J., Adams P.D. (2024). Guidelines for minimal information on cellular senescence experimentation in vivo. Cell.

[30] Jia G., Aroor A.R., Jia C., Sowers J.R. (2019). Endothelial cell senescence in aging-related vascular dysfunction. Biochim Biophys Acta Mol Basis Dis.

[31] Dobner S., Tóth F., de Rooij L.P.M.H. (2024). A high-resolution view of the heterogeneous aging endothelium. Angiogenesis.

[32] Guerrero A., Herranz N., Sun B. (2019). Cardiac glycosides are broad-spectrum senolytics. Nat Metab.

[33] Ambrosy A.P., Butler J., Ahmed A. (2014). The use of digoxin in patients with worsening chronic heart failure: reconsidering an old drug to reduce hospital admissions. J Am Coll Cardiol.

[34] Digitalis Investigation Group (1997). The effect of digoxin on mortality and morbidity in patients with heart failure. N Engl J Med.

[35] Abdul-Rahim A.H., MacIsaac R.L., Jhund P.S. (2016). Efficacy and safety of digoxin in patients with heart failure and reduced ejection fraction according to diabetes status: an analysis of the Digitalis Investigation Group (DIG) trial. Int J Cardiol.

[36] Patel N., Ju C., Macon C. (2016). Temporal trends of digoxin use in patients hospitalized with heart failure: analysis from the American Heart Association Get With the Guidelines-Heart Failure Registry. JACC Heart Fail.

[37] van Veldhuisen D.J., Rienstra M., Mosterd A. (2024). Efficacy and safety of low-dose digoxin in patients with heart failure. Rationale and design of the DECISION trial. Eur J Heart Fail.

[38] Yang Y., Lu X., Ning L., Ma S. (2024). Metformin decelerates aging clock in male monkeys. Cell.

[39] Fang J., Yang J., Wu X. (2018). Metformin alleviates human cellular aging by upregulating the endoplasmic reticulum glutathione peroxidase 7. Aging Cell.

[40] Barzilai N., Crandall J.P., Kritchevsky S.B., Espeland M.A. (2016). Metformin as a tool to target aging. Cell Metab.

[41] Katsuumi G., Shimizu I., Suda M. (2024). SGLT2 inhibition eliminates senescent cells and alleviates pathological aging. Nat Aging.

[42] Liu L., Ni Y.Q., Zhan J.K., Liu Y.S. (2021). The role of SGLT2 inhibitors in vascular aging. Aging Dis.

[43] Khemais-Benkhiat S., Belcastro E., Idris-Khodja N. (2020). Angiotensin II-induced redox-sensitive SGLT1 and 2 expression promotes high glucose-induced endothelial cell senescence. J Cell Mol Med.

[44] Dhakal B., Shiwakoti S., Park E.Y. (2023). SGLT2 inhibition ameliorates nano plastics-induced premature endothelial senescence and dysfunction. Sci Rep.

