# Supplementary material for: Endothelial Senescence Drives Deleterious Endothelial-Adipocyte Cross-Talk in Patients With Heart Failure and Type 2 Diabetes
Source: JACC Basic Transl Sci. 2026 Apr 10;11(5):101527. doi: 10.1016/j.jacbts.2026.101527 (PMC13091929; doi:10.1016/j.jacbts.2026.101527)
Supplement: Supplemental Material 1 [file mmc1.docx]

|  | **Control (n=25)** |
| --- | --- |
| **Sociodemographic** |  |
| Age, *years* | 72 (12) |
| Male | 17 (68.0) |
| Ever smoked | 4 (16.0) |
| BMI, *kg/m^2^* | 26.0 (6.4) |
| **Disease severity** |  |
| LVEF, *%* | 57 (5) |
| HbA1c, *mmol/mol* | 39 (4) |
| NT-proBNP, *pg/ml* | 784 (1067) |
| **Comorbidities** |  |
| Atrial Fibrillation | 6 (24.0) |
| COPD | 1 (4.0) |
| HTN | 7 (28.0) |
| **Blood results** |  |
| Hb, *g/L* | 137 (17) |
| Na, *mmol/L* | 139 (3) |
| K, *mmol/L* | 4.0 (0.5) |
| eGFR, *ml/min/1.73 m^2^* | 77 (11) |
| **Medication** |  |
| ACEi / ARB / ARNI | 7 (28.0) |
| Beta-blocker | 4 (16.0) |
| MRA | 1 (4.0) |
| Aspirin | 2 (8.0) |
| Statin | 13 (52.0) |
| SGLT2i | 0 (0.0) |
| Metformin | 0 (0.0) |
| Insulin | 0 (0.0) |

**Supplementary table 1 – Demographic characteristics of control patients.**

Continuous data presented as mean with standard deviation. Categorial data presented as n (%). Abbreviations: angiotensin converting enzyme inhibitor (ACEi); angiotensin receptor blocker (ARB); angiotensin receptor neprilysin inhibitor (ARNI); body mass index (BMI); chronic obstructive pulmonary disease (COPD); estimated glomerular filtration rate (eGFR) glycated haemoglobin (HbA1C); haemoglobin (Hb); heart failure (HF); heart failure and type 2 diabetes mellitus (HFDM); hypertension (HTN); left ventricular ejection fraction (LVEF); mineralocorticoid receptor antagonist (MRA); potassium (K); sodium (Na); sodium glucose co-transporter 2 inhibitor (SGLT2i); standard deviation (SD)

**
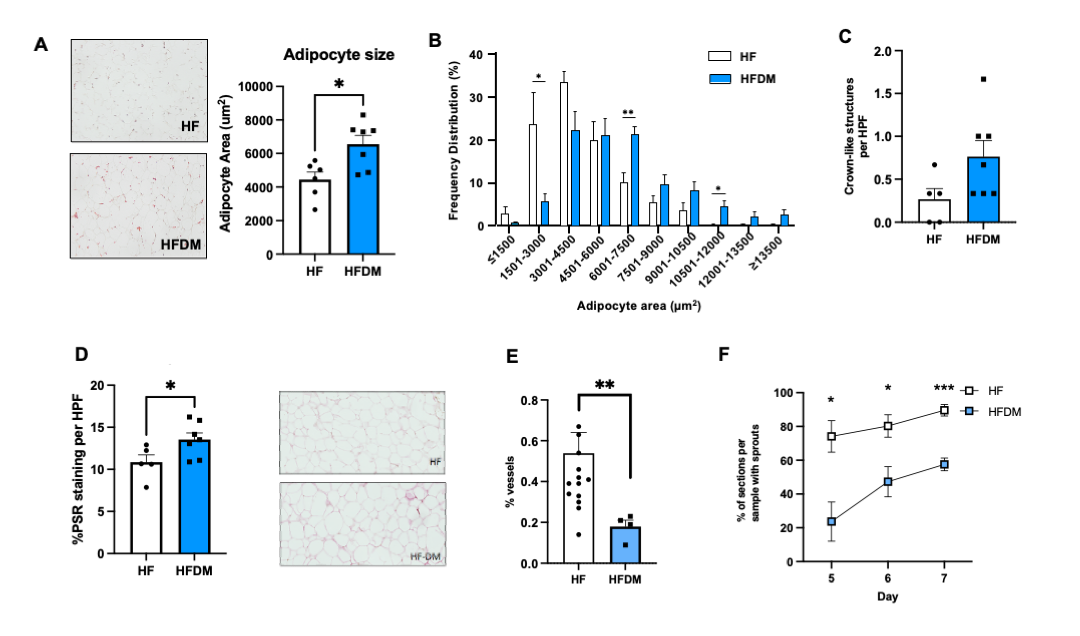
**

**Supplementary figure 1 – Whole fat analysis**

Panel A – Adipocyte size: showing mean adipocyte area (um^2^) in SAT calculated using ImageJ after haematoxylin and eosin staining between groups with illustrative microscopy. (n=6, 7). Panel B – Distribution of adipocyte size: showing histogram of adipocyte areas (um^2^) between groups (n=6, 7). Panel C – Mean number of crown-like structures per HPF between patient phenotypes (n=5, 7). Panel D – % Picrosirius red (PSR) staining in whole SAT with illustrative microscopy (n=5, 9). Panel E – Percentage of vessels within SAT measured using confocal microscopy. Panel F - Sprout forming assay: mean number of new endothelial cell sprouts per 1mm^3^ piece of dissected SAT embedded within a fibrin matrix over 7 days, minimum of n=20 per patient sample (n=15,5). * p<0.05, ** p<0.01, *** p<0.001


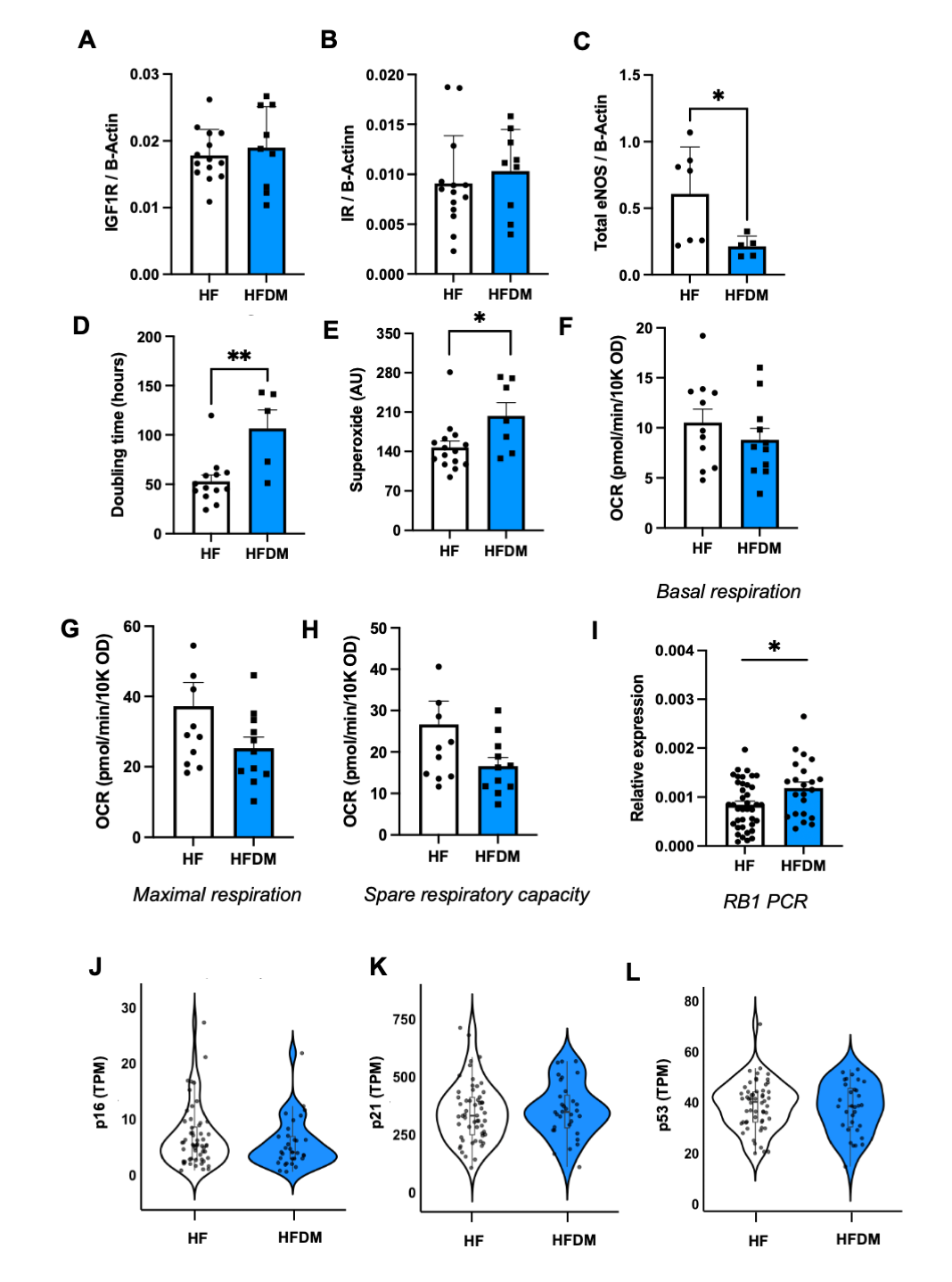


**Supplementary figure 2 – SATMVEC phenotyping**

Panel A: Protein expression of SATMVEC IGF1R (n=14,9) ; Panel B: Protein expression of SATMVEC IR (n=14,9) ; Panel C: Protein expression of SATMVEC Total eNOS (n=7,5) ; Panel D: SATMVEC population doubling time (n=13,5); Panel E: Superoxide production from SATMVEC (n=15,6); Panel F: SATMVEC basal mitochondrial oxidative consumption rate (n=12, 11), Panel G: SATMVEC maximal mitochondrial oxidative consumption rate (n=12, 11), Panel H: SATMVEC spare mitochondrial respiratory capacity (n=12, 11). Panel I: mRNA expression of RB1 in SATMVEC quantified with RT-qPCR(n=39, 22). Panel J: mRNA expression of p16 in SATMVEC plotted with jittered data points (n=56, 30); Panel K: mRNA expression of p21 in SATMVEC plotted with jittered data points (n=56, 30); Panel L: mRNA expression of p53 in SATMVEC plotted with jittered data points (n=56, 30). Data are presented as mean ± SEM with individual data points representing biological replicates, unless otherwise indicated. Violin plots show the distribution of individual values. P values for comparisons between HF and HFDM groups were calculated using Student’s t-test or Mann–Whitney U test, as appropriate based on data distribution. * p<0.05, ** p<0.01, *** p<0.001.


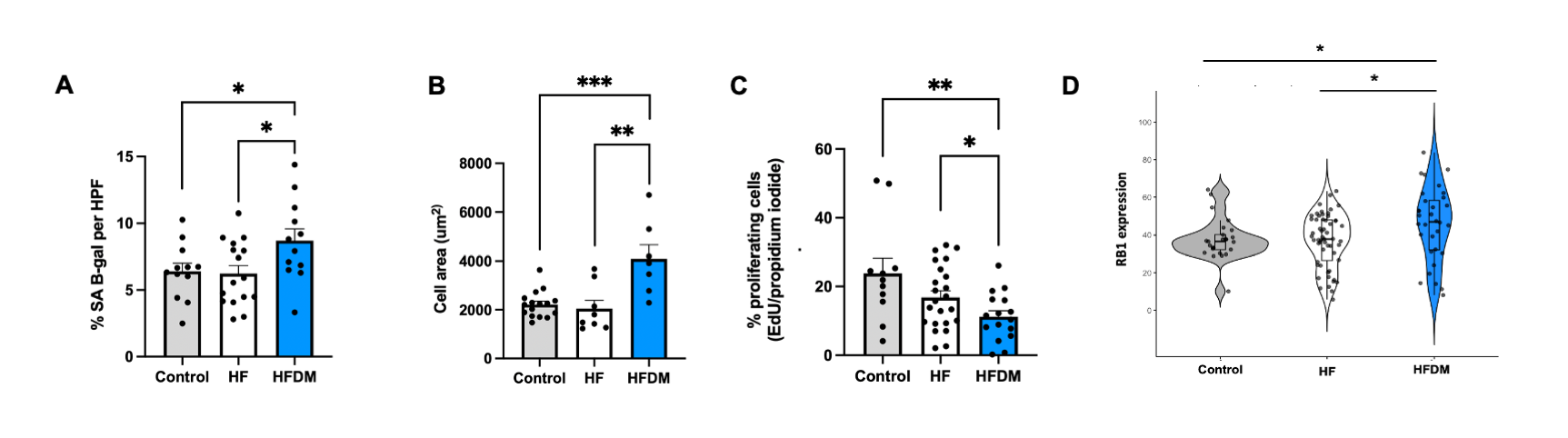


***Supplementary figure 3 – Markers of SATMVEC senescence in control patients compared to HF and HFDM patients.*** *Panel A: SATMVEC senescence histochemical senescence associated β-galactosidase staining with illustrative phase and fluorescent imaging (n=12, 16, 12), Panel B: SATMVEC cell size (n=16, 8, 7). Panel C: SATMVEC proliferation using Click-IT EdU imaging kit (n = 11, 19, 14).* Data are presented as mean ± SEM with individual data points representing biological replicates, unless otherwise indicated. ** p<0.05, ** p<0.01, *** p<0.001*

***Supplementary methods***

***Whole subcutaneous adipose tissue analysis***

*Histology*

After incubating for 24-hours in 4% paraformaldehyde (PFA) at 4°C, a sample of adipose tissue was dehydrated and paraffin embedded. Ten micrometre sections through the body of the tissue were collected and mounted onto glass slides. Samples were stained using haematoxylin and eosin (for tissue morphology, adipocyte size and crown like structures), and picrosirius red (for collagen deposition).[1] Three separate images were taken for analysis of each sample. The adipocyte size of every cell was measured using ImageJ to allow for the mapping of adipocyte size distribution, and the mean size for each sample was recorded. The number of crown-like structures per HPF was counted in three separate areas for each sample by two independent assessors (KB and OIB). The amount of red staining was quantified using thresholding in ImageJ.

*Vascular Density*

Whole tissue samples were washed in phosphate buffered saline (PBS), placed into 1% PFA for 2 hours at room temperature, then washed in PBS. Samples were incubated overnight with lectin from Ulex europaeus Alexa Fluor 594, diluted 1:100 in 5% BSA in PBS at 4°C. Following a further wash in PBS, samples were then incubated with HCS LipidTOX (Thermo Fisher Scientific), diluted 1:200 in PBS for 20 minutes at room temperature. Whole tissue sections were mounted onto slides using a silicone spacer with Prolong Gold. Slides were imaged using confocal microscopy, and the vascular density (the proportion of each image stained with lectin), was quantified using thresholding in ImageJ.

*Subcutaneous adipose tissue endothelial sprouting*

Angiogenesis assays from adipose tissue were performed using a modified technique as previously described. [2] In sterile conditions, any surface blood vessels were dissected from the adipose tissue sample, before it was cut into pieces no bigger than 1mm^3^. For each sample, at least 20 sections were embedded into a fibrin matrix. The fibrin matrix was achieved by combining 12.5ul of 50 U/ml thrombin (Sigma-Aldrich T-3399) with 500µl of a mix containing 4 U/ml aprotinin (Sigma-Aldrich A-1153) and 2 mg/ml fibrinogen type 1 (Sigma-Aldrich F-8630), and adding a piece of adipose tissue into the centre of the well. The plates were then incubated at room temperature for 20 minutes, and then at 37°C for a further 20 minutes to allow the matrix to gel. One millilitre of ECGM MV media (Promocell) was then carefully pipetted onto the top of each well, and plates were cultured for up to 7 days. The media was discarded and replaced every other day throughout the culture period. Each day, the samples were imaged at 4x magnification on Olympus florescent microscope CKX41 and the number of endothelial sprouts coming from each piece of fat was counted. For each sample, the average number of sprouts per section was calculated, as well as the number of sections (derived from the original sample) which had sprouted*.*

***Assessment of endothelial cell phenotype***

*RNA sequencing*

RNA was isolated from SATMVEC at P2 using TRIzol reagent (Invitrogen, USA) following the manufacturer’s protocol and concentration measured using a Nanodrop (Thermo Fisher Scientific). [20] RNA sequencing (RNA-seq) was performed using libraries constructed from 600ng RNA per sample by Novogene (Cambridge, UK). Raw FASTQ files were trimmed using TrimGalore to remove low quality reads and overrepresented sequences. A reference human genome was retrieved from the Ensembl genome database (GRCh38.p14). STAR aligner was then used to align the trimmed sequences to the reference human genome. Once aligned, raw read counts were calculated using FeatureCounts in R. Differentially expressed genes (DEGs) were identified using DESeq2 package (v1.40) and expressed as transcripts per million. As samples were sequenced at different time points, the sva package (v3.54) was used to adjust batch effects. Analysis was hypothesis testing rather than hypothesis generating, and Bonferroni adjustment was performed for canonical senescence genes of interest (p16, p21, p53 and RB1). A Bonferroni-adjusted P-value <0.05 was deemed statistically significant. R software version 4.3.0 (R Core Team, 2021) was used for analysis.

*Endothelial cell proliferation*

SATMVECs (P3-4) were seeded at a density of 2x10^4^ cells per well of a 24-well plate, and left overnight in full media, and proliferation assessed using the commercially available Click-IT Edu imaging kit as per manufacturer’s instructions. Nuclei were counterstained using propidium iodide staining solution (Cell Signaling, excitation and emission maxima 535/617 nm). Proliferating cells were counted and expressed as a % of total cells within each high-powered field (average of 4 regions per well).

*Endothelial cell tube formation*

SATMVEC were plated at 1x10^5^ cells (in one mL of media) per well of a 24well plate containing 160µl/well of Matrigel (Corning). After incubation at 37°C for 4 hours, the number of tubes in 5 different areas of the well were imaged using bright field phase contrast microscopy on the Incucyte Zoom, and the number of complete tubes per HPF counted. Results were verified by two independent blinded assessors (KB and OIB).

*Senesence associated β-galactosidase staining*

SATMVEC senescence associated β-galactosidase (SA-β-gal) was measured using the CellEvent Senescence Green Detection Kit as per manufacturer’s instructions. Cells were imaged at 10x magnification using the Incucyte Zoom, the amount of green staining was quantified using ImageJ, and expressed as a percentage of total cell coverage. Results were verified by two independent assessors (KB and OIB) blind to study group.

*High-throughput respirometry using Seahorse Real-Time Cell Metabolic Analysis*

To measure mitochondrial respiration SATMVECs were subjected to Seahorse XF Cell Mito Stress Test using the Seahorse XF Cell Mito Stress Test Kit on a Seahorse XFe96 Analyzer (Agilent Technologies) according to the manufacturer’s instructions. SATMVEC were plated at 3x10^4^ cells per well of a 96-well plate (each sample was run with 4-5 replicates) and left overnight in full MV media. Cells were washed in PBS before being left in Seahorse XF DMEM media containing 1 mM pyruvate, 2 mM glutamine, and 10 mM glucose for 1 hour prior to the assay. A total of three basal oxygen consumption rate (OCR) measurements were recorded prior to the injection of 1.5µM oligomycin to each well and three further measurements were made. The decreased level of OCR represented oligomycin-sensitive OCR due to its inhibition of ATP synthase (complex V). 1.0 µM FCCP (an uncoupling agent) was then injected and three further measurements were made. FCCP-stimulated OCR was used to calculate spare respiratory capacity, which was defined as the difference between maximal respiration and basal respiration. Finally, 0.5 µM rotenone and antimycin A (combination of complex I and III inhibitor) were added to each well. Three final measurements were then made. Cells were washed with PBS three times to remove assay media and were then fixed with 100µl of 4% PFA at room temperature for 20 minutes. Cells were stained with 2ug/mL Hoechst 33342 for 20 minutes before being washed with PBS for three times and fluorescence was then measured by Cytation5 imaging reader (excitation/emission 352/545nm). Data was then normalized to Hoechst staining and expressed as oxygen consumption rate (pmol O₂/min) per unit of Hoechst optical density (OD), representing mitochondrial oxygen consumption rate normalized to cell number.

*Endothelial cell secretome*

SATMVECs (P3) were plated into a T25 flask and left to grow to confluence in ECGM MV media. Once cells reached confluence, the media was removed, cells were washed using PBS, and 5mLs of ECGM MV media without supplement was added to each sample. Cells were incubated in normal conditions (37°C, 5% CO_2_) for 24 hours, after which time the conditioned media (CM) was collected, and stored at -80°C.

The senescence associated secretory phenotype of SATMVEC was assessed by analysing CM using the MAGPIX Cytokine 10-Plex Human Panel (Thermofisher). Quantitative protein expression was then performed using xPONENT™ software (Thermofisher). Target bead count was set at ≥200 beads. The assay standard curve was produced using 5 parameters logistic regression. All samples were run in duplicate. CM protein content was standardised to total protein quantified by BCA assay. To reduce intra-assay variability, only results with ≤10% CV of replicates were included in analyses.

*SATMVEC Population Doubling*

At each passage, cells were counted to allow calculation of their population doubling time. Once confluent (P1), cells were gently dissociated from the plate using TrypLE Express (Thermofisher). Once in suspension, the TrypLE was neutralised by adding 2x volume of full MV media. Using an electronic cell counter (Merck Scepter™ 2.0 cell counter), the number of cells per ml was elucidated, and the total number of cells was calculated. Cells were then passaged at a maximum ratio of 1:6, and maintained at 37°C 5% CO_2_. Once confluent again (P2), the total number of cells were again counted using an electronic cell counter. The cell doubling time was then calculated using the formula: doubling time = (duration X log(2))/((log (final concentration)-log(initial concentration)). An online calculator is available at: <https://doubling-time.com/compute.php>

***Adipocyte culture, co-culture set up and phenotyping***

Human white subcutaneous pre-adipocytes at P2 (Lonza) from a single healthy Caucasian donor were cultured until confluent using PGM-2 media in each well of a 24-well co-culture plate (Appleton Woods) for RNA extraction and NBD-glucose uptake and 12-well co-culture plate (Appleton Woods) for protein extraction. Cells were maintained at 37°C 5% CO_2_ with a full media change every three days. Once confluent, cells were differentiated using PDM media. After addition of the differentiation media, cells were left to incubate for 10 days (without media changes), to fully differentiate. Differentiation was monitored daily using light phase microscopy.

On day 6, transwell inserts (0.4uM membrane) were seeded with SATMVEC (P4) at a density of 5000 cells per insert for 24-well inserts and 15000 cells per insert for 12-well inserts and grown in a separate plate in EGCM-MV media (Promocell) to confluence. On day 10, when pre-adipocytes were fully differentiated, half the PDM media was removed from each well and transwell inserts containing confluent SATMVEC in their own media (ECGM-MV, Promocell) were added to each well. Cells were left in co-culture for 24 hours. After this time, the transwell inserts were removed, and the function of the adipocytes were assessed.

For digoxin treated experiments, SATMVEC insert media was changed to 1nM digoxin ECGM MV media or fresh ECGM MV media (as a control) on day 9 of adipocyte culture and were left for 24 hours. SATMVEC wells and inserts were then washed twice in PBS prior to being added to adipocyte wells and left for a further 24 hours before adipocyte functional assays.

*NBD-Glucose uptake*

After 24-hours in co-culture, 2-NBD-glucose was added to PDM at a final concentration of 20µM. Following a 30 minute incubation, the cells were washed in PBS, and fixed using 4% PFA for 15min at room temperature. Cells were then imaged (Incucyte ZOOM), with glucose shown as fluorescent green (excitation/emission 465/540nm). Four images were taken for each sample, and an average recorded. The level of glucose uptake was quantified using thresholding in ImageJ. To ensure comparability across samples, identical threshold settings were applied to all images in ImageJ to define fluorescent signal above background. Results were verified by two independent assessors (KB and OIB) blinded to study group.

*Cell surface biotinylation*

For cell surface biotinylation, differentiated (pre-) adipocytes were co-cultured with SATMVEC for 24 h in a 12-well co-culture plate (Appleton Woods) as described above. For experimentation, adipocytes were washed twice with M199 (Gibco) supplemented with 1% AAS, 1 mM Sodium Pyruvate, 10 mM HEPES (all Merck) and placed on ice. Cells were washed once in ice-cold PBS containing Ca2+ and Mg2+ (Gibco) prior to addition of 1 mM EZ-link sulfo-NHS-SS biotin (Thermo-Fisher) for 30 min on ice. To quench excess biotin, cells were washed twice for 10 min with STOP-solution (100 mM Tris, pH 7.4/ 1 mM Ca2+ & 1 mM Mg2+; all Merck), followed by one wash in PBS containing Ca2+ and Mg2+ to remove excess Tris. Cells were lysed in cell extraction buffer (Invitrogen). Protein concentration was determined using a BCA-assay kit (Thermo-Fisher). Biotinylated proteins from 100 mg lysate were concentrated using magnetic streptavidin beads (Pierce) following the manufacturer’s suggestions. 10 mg whole cell lysate and concentrated proteins were analysed by Western blotting. Membranes were probed with antibodies for GLUT4 (1:500, Cell Signal Technology, clone 1F8) and HSP90 (4 ng/mL, Santa Cruz Biotechnology, clone F-8).

***General techniques***

*Quantification of gene expression*

RNA was extracted from whole tissue/cultured adipocytes using the Monarch Total RNA Miniprep Kit. The concentration of RNA in each sample was elucidated using a Nanodrop (Thermo Fisher Scientific). cDNA was reverse transcribed using the Lunascript RT Supermix Kit, according to the manufacturer’s instructions. Real time quantitative PCR (RT-qPCR) was performed using a Roche LightCycler 480 Instrument II, using SYBR Green PCR Master Mix (Bio-Rad, 1725120) and relevant primers. All samples were run in duplicate. RT-qPCR was analysed using the 2-ΔCt method, with the gene of interest expressed relative to GAPDH/HPRT1 where appropriate.

References

1. Junqueira LC, Bignolas G, Brentani RR. Picrosirius staining plus polarization microscopy, a specific method for collagen detection in tissue sections. *Histochem J*. 1979; **11**: 447–55.

2. Luk C, Bridge KI, Warmke N, Simmons KJ, Drozd M, Moran A, MacCannell AD V, Cheng CW, Straw S, Scragg JL, Smith J, Ozber CH, Wilkinson CG, Skromna A, Makava N, Prag HA, Simon Futers T, Brown OI, Bruns A-F, et al. Paracrine role of endothelial IGF-1 receptor in depot-specific adipose tissue adaptation in male mice. *Nat Commun*. 2025; **16**: 170.
